# Assessment of cigarette smoke particle deposition within the Vitrocell® exposure module using quartz crystal microbalances

**DOI:** 10.1186/1752-153X-7-50

**Published:** 2013-03-12

**Authors:** Jason Adamson, David Thorne, Annette Dalrymple, Debbie Dillon, Clive Meredith

**Affiliations:** 1 British American Tobacco, Group R&D, Southampton, SO15 8TL, UK

**Keywords:** Dosimetry, Particle deposition, QCM, Quartz crystal microbalance, Tobacco smoke, *In vitro* whole smoke exposure systems, Vitrocell®

## Abstract

**Background:**

Cigarette smoking is a cause of a variety of serious diseases, and to understand the toxicological impact of tobacco smoke *in vitro*, whole smoke exposure systems can be used. One of the main challenges of the different whole smoke exposure systems that are commercially available is that they dilute and deliver smoke in different ways, limiting/restricting the cross-comparison of biological responses. This is where dosimetry – dose quantification – can play a key role in data comparison. Quartz crystal microbalance (QCM) technology has been put forward as one such tool to quantify smoke particle deposition *in vitro*, in real-time.

**Results:**

Using four identical QCMs, installed into the Vitrocell® mammalian 6/4 CF Stainless exposure module, we were able to quantify deposited smoke particle deposition, generated and diluted by a Vitrocell® VC 10 Smoking Robot. At diluting airflows 0.5-4.0 L/min and vacuum flow rate 5 ml/min/well through the exposure module, mean particle deposition was in the range 8.65 ± 1.51 μg/cm^2^-0.72 ± 0.13 μg/cm^2^. Additionally, the effect of varying vacuum flow rate on particle deposition was assessed from 5 ml/min/well - 100 ml/min/well. Mean deposited mass for all four airflows tested per vacuum decreased as vacuum rate was increased: mean deposition was 3.79, 2.75, 1.56 and 1.09 μg/cm^2^ at vacuum rates of 5, 10, 50 and 100 ml/min/well respectively.

**Conclusions:**

QCMs within the Vitrocell® exposure module have demonstrated applicability at defining particle dose ranges at various experimental conditions. This tool will prove useful for users of the Vitrocell® system for dose–response determination and QC purposes.

## Background

Whole smoke exposure systems have been used to assess the toxicity/biological effect of tobacco smoke *in vitro *[1-8]. Generally these exposure systems comprise of a smoking machine which dilutes and delivers mainstream cigarette smoke to an exposure chamber which can contain different cell cultures, usually supported at the air-liquid interface (ALI). Some whole smoke exposure systems are simple, consisting of a small chamber containing a cell culture plate where mainstream cigarette smoke is delivered via a vacuum pump and/or fan [4,8]. Other systems are more sophisticated. Such commercial examples are supplied by Borgwaldt KC, Germany [9], Burghart Tabaktechnik, Germany [10,11] and Vitrocell® Systems, Germany [3,12]. Commercially available exposure systems have been coupled to exposure chambers/modules, originating from British American Tobacco Group Research & Development, UK [2,13], CULTEX® Laboratories, Germany [14] and Vitrocell® Systems. These exposure chambers/modules are not only used for tobacco smoke exposure but may be employed to assess other inhalable aerosols/substances [15-18].

Exposing cell cultures to a whole smoke aerosol at the ALI is technically challenging. This is mainly due to the complexity of concentrated tobacco smoke aerosol, the requirement to dilute smoke homogeneously with air, and the need for highly controlled experimental conditions to achieve reproducible and comparable results [19]. Importantly, what a well characterised system offers is confidence in the exposure set-up and experimental conditions that such testing requires.

Of the commercially available whole smoke exposure systems, one of the main challenges is that they dilute and deliver smoke in different ways, which often limits/restricts the cross-comparison of biological responses from these various platforms. However, dosimetry tools will play a key role in aiding data cross-comparison. Dosimetry, in the context of inhalation toxicity testing, is the accurate determination of test article dose (whether that be whole smoke or any other inhalable aerosol) reaching the target cells, and is one of the key components in the establishment of a reliable dose–response [19].

Currently, there are a limited number of (characterised/published) tools available to quantify tobacco smoke dose accurately; this may be due to the smoke being partitioned into two phases meaning each tool has a specific requirement and design. The physical form of tobacco smoke is spherical liquid droplet particles suspended in a gas vapour. This smoke comprises thousands of chemicals and toxicants distributed across both physical states (particle and gas); the particulate phase approximating 5% of whole smoke by weight [5]. Thus the primary challenge where dosimetry is concerned is that one tool will be unable to quantify both phases simultaneously – tools will either quantify the gas vapour or particulate phase. For example, gas analysers can assess simple gas components of smoke such as carbon monoxide [20], or time-of-flight mass spectroscopy (TOF-MS) could be used in the future to measure more complex species in tobacco smoke such as acetaldehyde, acetone, benzene and toluene [21]. In the case of the particle phase, quantification can be by chemical elution or the use of a microbalance [22]. Optical tar measurements can be recorded using small light scattering photometer devices [3] and these are particulate phase tools rather than gas/vapour tools, as they scan the particles suspended within the gas.

Of the tools available to measure smoke dose, those with the ability to measure in real-time improve the reliability of the measured dose–response relationship [23]. Currently, a tool which is characterised and enables real-time quantification of smoke particle deposition per unit area is the quartz crystal microbalance (QCM). This tool has been utilised in the field of *in vitro* toxicity testing of airborne nanoparticles/chemicals [19,23] and for the first time with diluted whole smoke *in vitro*[22].

**Figure 1:**
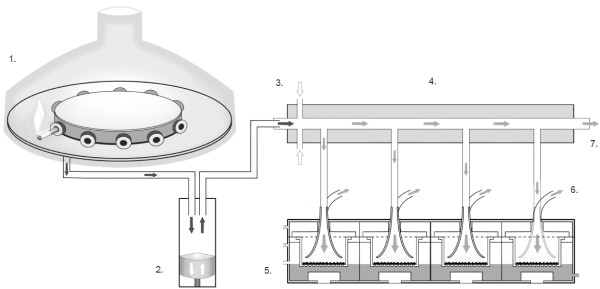
**The Vitrocell VC 10 Smoking Robot and mammalian exposure module (6/4 CF Stainless) – a schematic cross-section.** ([1]) Smoking robot carousel where cigarettes are loaded and smoked, enclosed in a ventilation hood. ([2]) Piston/syringe which draws and delivers ISO (35 ml) or Health Canada Intense (55 ml) mainstream cigarette smoke. ([3]) Air jets add continuous diluting air perpendicular to the mainstream smoke in the range 0.2-12 L/min; rates are set and maintained by mass flow controllers. ([4]) Dilution, transit and delivery of whole smoke occurs in the dilution bar, multiple parallel bars make up the dilution system. ([5]) Isolated cell culture inserts are supported and exposed to diluted whole smoke at the ALI in a module which docks under the dilution system; culture inserts can be removed and replaced with QCMs. ([6]) A vacuum (5–100 ml/min/well) is applied to the module which draws the diluted smoke from the dilution bar into the module via the ‘trumpet’ inlets. ([7]) Due to continuous diluting airflow, smoke remaining within the dilution system transits to exhaust away from the module.

**Figure 2:**
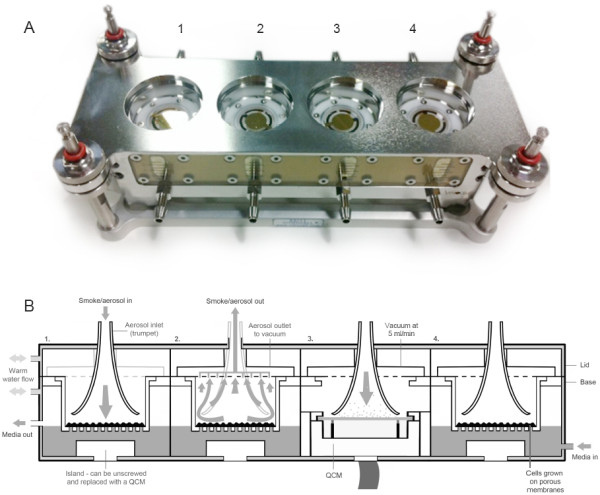
**The Vitrocell mammalian exposure module (6/4 CF Stainless).** [**A**] Top view of the module base (lid removed) looking into the four separated wells where cell culture inserts would usually sit and be exposed to smoke, but where four identical QCM units have been installed, 1–4 (left-right). [**B**] A schematic cross-section of the module base and lid depicting how smoke is delivered to cell culture inserts/QCM. This illustration shows a very slight difference in the heights of the crystal surface and a typical cell exposed on a porous membrane; in our set-up the QCM is 1.5 mm higher than an exposed cell surface would be. However, the trumpet height can (and should) be adjusted to compensate for this when exposing cells and QCMs concurrently so that the gap distance is the same (2 mm in this case between crystal and trumpet).

In this study, a Vitrocell VC 10 Smoking Robot was used to generate and dilute cigarette smoke (Figure 1). Smoke was delivered to a Vitrocell mammalian 6/4 CF Stainless exposure module containing four identical QCM units (one each into the separate culture wells) (Figure 2) and the real-time assessment of dose and dose-range quantification of deposited smoke particles determined. The QCM experiments detailed have also enabled the parameter of airflow and vacuum rate, which are used for VC 10 smoke dilution, to be converted into simple and comparable units of mass per surface area. These units allow a better understanding of dose and will facilitate the future cross-comparison of biological data.

Microbalances (QCMs) have been used for a variety of monitoring and quantification applications over the years. Such applications have included measurement of water pollution [24], detection of microscopic entities such as virus nanoparticles [25], quantification of smoke types including outdoor tobacco smoke [26] and to assess occupational exposure to blood and bone cautery smoke derived from orthopaedic surgery [27].

In this study we present a characterised dosimetry tool utilised to quantify particle deposition *in vitro* from the Vitrocell VC 10 Smoking Robot; QCM units were installed into the Vitrocell mammalian exposure module (Figure 2). Smoke from the VC 10 was diluted by adjusting the airflow supplied to the dilution system (Figure 1 position 3) and four airflows were tested in the range 0.5-4.0 L/min, which is consistent with other published exposures using the VC 10 [12]. Additionally, the effect of varying vacuum flow rate on particle deposition was assessed from 5 ml/min/well up to 100 ml/min/well.

## Results

**Figure 3:**
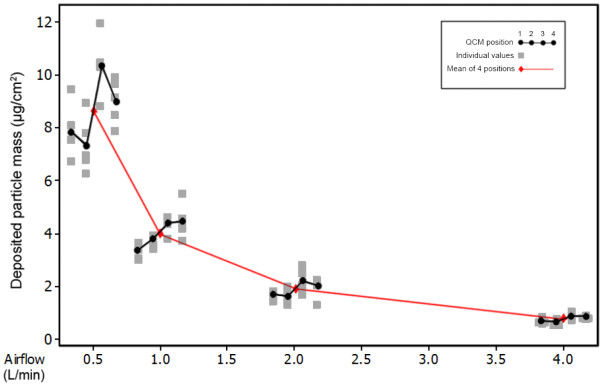
**Dose–response of QCM quantified whole smoke particle deposition in the Vitrocell 6/4 CF Stainless module.** A multi-vari chart with individual values showing deposited particle mass for the 4 QCM positions (1–4, left-right) at diluting airflows ranging 0.5-4.0 L/min and sampling from the dilution system at 5 ml/min/well vacuum through the module (n = 5/QCM position/airflow).

Using four microbalances installed into the Vitrocell mammalian 6/4 CF Stainless exposure module we were able to quantify deposited smoke particle deposition, generated and diluted by a Vitrocell VC 10 Smoking Robot. At the standard (supplier recommended) vacuum flow rate of 5 ml/min/well, mean particle deposition (mean of all 4 well positions repeated 5 times) was in the range 8.65 ± 1.51 μg/cm^2^-0.72 ± 0.13 μg/cm^2^ in the airflow range 0.5-4.0 L/min. Across the module, there were observed patterns/gradients of deposition for positions 1–4 (left-right) (Figure 3). For example, at 1.0 L/min there was ascending deposition across the module, and at 0.5 L/min distribution was varied in an ‘S’ shaped formation (Figure 3). At all airflows tested there were significant differences between all 4 QCM positions, as determined by ANOVA testing (p = <0.05). At the most dilute airflow tested, 4.0 L/min, the four positions look the most equivalent. At 2.0 L/min the means diverge but positions 1 and 2 (proximal to smoke entry in the module) were paired; at 1.0 L/min they diverge further, this time with positions 3 and 4 (distal to smoke entry in the module) paired. At the most concentrated dose of 0.5 L/min the divergence is great enough that the significance between them gave a p-value of <0.01.

**Figure 4:**
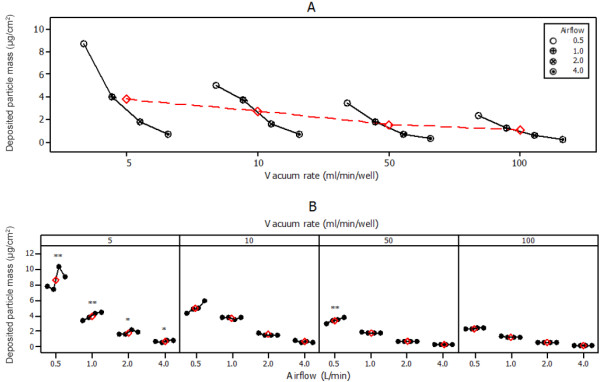
**QCM quantified whole smoke particle deposition in the Vitrocell module at various airflow (L/min) and vacuum (ml/min/well) rates.** [**A**] A multi-vari chart showing deposition within the dose range of diluting airflows 0.5-4.0 L/min and at vacuum rates of 5–100 ml/min/well through the module (mean of 4 QCM positions per airflow). [**B**] A multi-vari chart showing the regional deposition across the module (separated by vacuum). The 4 connected black dots represent QCM positions 1–4, left to right (Figure 2A). Asterisks indicate statistically significant differences in deposition across/between module positions 1–4 as determined by one-way ANOVA: * denotes a statistically significant difference between positions, p = 0.05-0.01, ** denotes a statistically significant difference between positions, p = <0.01. For 5 ml/min/well vacuum, data sets were n = 5/QCM position/airflow; for 10, 50 and 100 ml/min/well vacuum, data sets were all n = 3/QCM position/airflow. For both charts, red diamonds indicate the mean deposited particle mass for all 4 airflows tested at each vacuum rate.

To build upon our understanding of how particle deposition changes with vacuum rate, the airflow experiments were repeated three more times (at the same airflows) but the vacuum was increased to 10, 50 and 100 ml/min/well. Figure 4A shows again the mean dose range at 5 ml/min/well (far left) and illustrates when vacuum is increased up to 100 ml/min/well that the overall dose range 0.5-4.0 L/min gradually decreases. For example, as Table 1 shows, at 5 ml/min/well for 0.5 L/min airflow mean deposited mass was 8.65 ± 1.51 μg/cm^2^, which decreased to 5.04 ± 1.19 μg/cm^2^ at 10 ml/min/well, then 3.41 ± 0.31 μg/cm^2^ at 50 ml/min/well and finally 2.34 ± 0.26 μg/cm^2^ at 100 ml/min/well. Overall, between vacuum rates of 5 and 100, the decrease in mean deposited mass for 0.5 L/min airflow was 6.31 μg/cm^2^. At the lower concentration of 4.0 L/min the dose range was 0.72 ± 0.13 μg/cm^2^-0.21 ± 0.07 μg/cm^2^ which resulted in a decrease in mean deposited mass of 0.51 μg/cm^2^. The data presented in Figure 4A illustrates the reduction in dose–response relationship across the vacuum rates tested (the overall response getting smaller as vacuum rate increased). These data are the means of the four QCM positions and therefore do not give an indication of deposition uniformity across QCM positions. Thus Figure 4B shows the same dose–response data with the pattern of deposition across the module. In each case, the 4 connected dots represent deposited particle mass across positions 1–4, left-right (Figure 2A), n = 5 for 5 ml/min/well, n = 3 for 10–100 ml/min/well. As discussed previously, at 5 ml/min/well there were statistically significant differences between the positions at all airflows tested (Figure 4B). As vacuum rate was increased to 10 ml/min/well the distribution improved and there were no significant differences in QCM position (despite at 0.5 L/min the trend suggesting there is an ascending gradient across the means). At 50 ml/min/well, deposition was observed to be uniform at airflow rates 1.0-4.0 L/min. At 0.5 L/min airflow and 50 ml/min/well vacuum there was a significant difference between positions, and this was due to the extremely tight data set and low standard deviation for each position (Table 1). Finally, at the highest vacuum rate of 100 ml/min/well, at all airflows tested there were no differences between positions; Figure 4B clearly shows uniform distribution across the module for each airflow/vacuum combination.

**Table 1:** A matrix of mean particle deposited mass values at various diluting airflow rates (from the VC 10 Smoking Robot) and vacuum rates (applied to the Vitrocell exposure module)

**Airflow**	**Vacuum rate (ml/min/well)**			
(L/min)	5	10	50	100
0.5	8.65 ± 1.51	5.04 ± 1.19	3.41 ± 0.31	2.34 ± 0.26
1.0	3.98 ± 0.61	3.70 ± 0.30	1.79 ± 0.21	1.24 ± 0.12
2.0	1.83 ± 0.39	1.57 ± 0.35	0.73 ± 0.09	0.56 ± 0.16
4.0	0.72 ± 0.13	0.67 ± 0.11	0.34 ± 0.02	0.21 ± 0.07

Values are the means ± SD (μg/cm^2^). For the 5 ml/min/well vacuum, each airflow data set was n = 20 (5 repeats/QCM position); for 10, 50 and 100 ml/min/well vacuum, each airflow data set was n = 12 (3 repeats/QCM position).

Analysis of variance (ANOVA) was used to test for significant differences across the QCM positions and is summarised in Table 2. Overall, at 5 ml/min/well there were significant differences at all the airflows tested. As vacuum rate was increased, there was a reduction in exposure module deposition and positional differences observed. At the highest vacuum rate of 100 ml/min/well there were no difference across the positions, exemplified by high p-values.

**Table 2:** Differences in particle deposition across/between positions 1–4 in the Vitrocell 6/4 CF Stainless module

**Airflow**	**Vacuum rate (ml/min/well)**			
(L/min)	5	10	50	100
0.5	0.001	0.451	0.000	0.966
1.0	0.002	0.568	0.940	0.711
2.0	0.044	0.587	0.950	0.997
4.0	0.012	0.130	0.639	0.992

(p-values determined by one-way ANOVA). For the 5 ml/min/well vacuum, each airflow data set was n = 20 (5 repeats/QCM position); for 10, 50 and 100 ml/min/well vacuum, each airflow data set was n = 12 (3 repeats/QCM position).

## Discussion

Cigarette smoking is a cause of a variety of serious diseases. Although the association between tobacco smoke and diseases such as chronic obstructive pulmonary disease (COPD) [28] and lung cancer [29] is known, many mechanisms of the disease are not. In part this is due to the fact that tobacco smoke is a concentrated, complex and dynamic aerosol made up of thousands of chemicals split between two physical states: gas and particle [5]. The most recent estimate on the number of chemicals is 5,600 individual smoke constituents [30] of which approximately 158 are understood to have toxicological properties [31].

To understand the toxicological and pathological impact tobacco smoke has on living cells and tissues, *in vitro* exposure systems can be used. Traditionally, biological testing of tobacco smoke constituents has been more routine, involving exposure to particulate matter extracted in a solvent or buffer, and applied to cells under a submerged condition [32]. Although these submerged lung culture assessments are quick and comparatively cheap (compared to whole smoke exposure systems), they do not reflect the physiological condition of the lung organ and its epithelial cells which sit at the blood-gas barrier and are exposed to air and other inhalable substances at the ALI. In response to this challenge, *in vitro* exposure systems have been developed to enable lung cell culture and whole smoke dose interactions at the ALI, better reflecting human exposure. There are a number of smoking machines and exposure chambers/modules which house cell cultures that can be used for such research, that are commercially available. These whole smoke exposure systems are of even greater importance with the recommendation that scientists working in tobacco research switch from liquid (submerged) to ALI exposed lung cell cultures *in vitro *[23]. But with the diversity of available machines and exposure chambers comes a caveat: how to interpret the biological data generated from various exposure systems? This is where dosimetry, or more specifically the tools used to define dose, will have significant value. Dose tools are of increasing importance to scientists and regulators as they will enable consistent interpretation of results and quick cross-comparison of biological end-points for defined doses of smoke [33].

The VC 10 is a complex system and as of yet there is very limited published information on its utility for whole smoke assessment; this is in contrast to similar apparatus where much more literature is available, especially on system characterisation (dilution principles, aerosol dynamics, physico-chemical measurements, system smoke losses, particle impaction/deposition and smoke interactions in the exposure chamber/module) [9-11,20,34,35]. As such, it is unclear what effects certain VC 10 variables/settings have on dose, such as jet diameter, airflow and vacuum rate. Therefore, what we present here is valuable information for other Vitrocell system users and users of related or similar systems.

In this study we used QCMs as a dose tool to quantify smoke particulate deposition *in vitro*. Four identical microbalance units were installed into the Vitrocell mammalian exposure module (6/4 CF Stainless) and quantified deposited particle matter from diluted whole smoke generated from the Vitrocell VC 10. With diluting flow rates of 0.5-4.0 L/min and vacuum rate through the module at 5 ml/min/well, the range of deposited mass detected was 8.65-0.72 μg/cm^2^ (Figure 3). At all airflow rates tested, there were significant differences in the distribution of particle deposition across the four QCM positions (Table 2): deposition was not uniform left-right despite the vacuum rate being set and maintained for each of the four wells, and smoke being sampled from the same dilution bar. Statistical analysis of experimental variables demonstrated there was no sensitivity to any of the remote oscillating units associated with each QCM (data not shown), thus irregular deposition appears to be originating from the dilution bar and the mixing of smoke within it. This has also been noted by other users of linear exposure modules (such as the CULTEX module) where a large variation in response was observed as a certain limitation of the module’s linear arrangement, thus a concentration gradient should be taken into consideration [16]. As the vacuum rate was increased, particle deposition uniformity across the chamber demonstrably improved (Figure 4B and Table 2). We theorise that an increase in vacuum rate increases the level of turbulence along the transit path of smoke, resulting in improved smoke mixing, which ultimately delivers a homogeneous test article to the surface of the QCM. In addition, smoke dilution/flow rate and transit (aging) may also influence the quality of the smoke deposition. Typically, as smoke ages it is evaporation which causes a significant proportion of the reduction in particle size, for example as semi-volatile nicotine transitions wholly from the particulate phase to the vapour phase. However, in the case of the VC 10 which has an 8 second delivery time from syringe to dilution system, coagulation would occur during puffing in the syringe, but the particles would stabilise during dilution and shrink back to a consistent size. In this study we did not assess particle size distribution, however we have an indication of this from a similar smoking machine used for *in vitro* exposure to cigarette smoke: the Borgwaldt RM20S [9]. In this system the transit length of smoke is over double that of the VC 10 at 3.4 m, and smoke losses prior to delivery at the exposure module were 47% in total, with 16% particle deposition by mass within the chamber [9]. The volume median diameter was recorded at 422.9 ± 2.5 nm at the syringe of the RM20S (undiluted), and 393.8 ± 10.4 nm at entry to the exposure chamber at a 1:60 dilution (smoke:air, v:v) [9].

Increased vacuum appears to improve the delivery/uniformity of smoke particles into the module well, however vacuum rates greater than 10–15 ml/min/well may not be favourable for biological testing, as increased flow may cause cellular stress. Certainly mammalian cell cultures which require a level of humidity and/or thin surface liquid for viability may not withstand increased flow rates. However, this may not be an issue for traditional genotoxicty tests such as the Ames assay; as well as being exposed to smoke in agar, prokaryotic cells are more robust than eukaryotic cells due to the presence of a cell wall, thus would be more resistant to these dehydrating flows. Indeed, vacuum levels up to 100 ml/min/well may be suitable for the Ames assay, but preliminary investigations would need to be conducted to confirm this.

In the standard set-up for mammalian exposure, a vacuum rate of 5 ml/min/well was used to sample smoke into the module. The vacuum rate of 5 ml/min is suggested as the optimum rate used for the Vitrocell set-up primarily to avoid dehydration to the cells [3,15]. In the similarly designed CULTEX module which also requires a vacuum to pull aerosol into the module (and can be used with the VC 10 [12]), flow rates have also been documented at 5 ml/min/well [16] and elsewhere at 8.3 ml/min/well (25 ml/min/module) [7,18]. Thus, to fully understand the effect of varying vacuum rate on smoke draw through the module we investigated rates greater than 5 ml/min/well. At the same four airflows previously tested, 0.5-4.0 L/min, we quantified particle deposition at 10, 50 and 100 ml/min/well. There are two observations in the vacuum study data that require comment: the airflow and vacuum combinations of 0.5 L/min and 10 ml/min/well, and 0.5 L/min and 50 ml/min/well. In the former, at 0.5 L/min diluting airflow and 10 ml/min/well vacuum, there appeared to be a significant ascending gradient in the mean values from positions 1–4 (Figure 4B) but no significant difference with a p-value of 0.451 (Table 2). The reason for this was that the standard deviation for this data set was relatively high at 5.04 ± 1.19 μg/cm^2^ (Table 1) thus the data range for all QCM positions overlapped with each other. This was in contrast to the latter observed combination of 0.5 L/min diluting airflow and 50 ml/min/well vacuum, where there appeared to be much less of an ascending gradient across positions 1–4 (or at least less than 0.5 L/min diluting airflow and 10 ml/min/well vacuum) (Figure 4B) but there was a significant difference with a p-value of <0.01 (Table 2). The reason for this was the standard deviation for this data set was comparatively low at 3.41 ± 0.31 μg/cm^2^ (Table 1), thus the data range for the 4 positions did not overlap. These experiments were repeated 3 times, and despite the combination of 0.5 L/min diluting airflow and 50 ml/min/well vacuum being interspersed randomly with the other combinations tested, the results were consistent. Without further testing it is unclear if this is simply because of this specific combination in the VC 10 set-up.

A final overall observation from the vacuum study was that as vacuum rate was increased, the overall dose range of deposition decreased (Figure 4A). The mean deposited mass values for all four airflows tested (Figure 4, red diamonds) was 3.79, 2.75, 1.56 and 1.09 μg/cm^2^ at increasing vacuum rates of 5, 10, 50 and 100 ml/min/well respectively. This was counter to our original hypothesis where we believed that increased vacuum rate would increase particle deposition – this was based on previous investigations (prior to the use of QCMs) demonstrating an increase in cytotoxicity with increased vacuum applied to the module. Thus we had hypothesised this because we believed more smoke was being sampled into the module at higher vacuum rates. The data obtained in this study indicated that particle matter deposition was in fact reduced due to the higher flow rates. Considering the size of particles present in tobacco smoke, deposition would occur predominantly through Brownian motion and diffusion, thus it is expected that a higher relative deposition would be achieved with a lower air flow through the dilution system; the same is true for a higher recorded deposition with a lower vacuum applied to the Vitrocell module. In addition to this, the gap distance between the trumpet and the surface of the exposed surface (crystal or cell culture) is important. In our set-up there was no difference in the heights of the four QCMs, and despite Figure 2 showing that there was a difference in the heights of the crystal surface and a typical cell exposed on a porous membrane, the height of the trumpets can be adjusted so that the gap distance is equivalent between the different surfaces (typically 2 mm in this case). This is important as larger gap distances would play a significant part in deposition; the wider the gap (and the smaller the particle size) the greater the chance particles will be lost around the trumpet and exhaust from the module without impacting on the surface. It was also observed that the increased draw through the module ultimately increased vapour phase transit and contact onto the crystal surface, resulting in a visual increase in yellow ‘material’ being removed during cleaning, despite the decrease in deposited mass overall. This again would explain our previous observation of an increase in cytotoxicity (observed in a continuous adenocarcinoma lung cell line (NCI-H292)) with increased vacuum, being driven by the vapour phase rather than the particulate phase. Only chemical elution and qualification will help to identify what was deposited on the QCM and help elucidate what proportions of smoke (vapour phase vs particulate phase) were increasing and decreasing with vacuum flow rates. These observations are important considerations for any users of the VC 10 and 6/4 CF Stainless module combination when designing exposure scenarios with varying vacuum flow rate, and the subsequent interpretation of biological data thereafter.

We have previously reported and characterised particulate deposition using a similar QCM tool [22], however this tool was used with a different whole smoke exposure system (Borgwaldt RM20S). For the first time, and exemplified by QCM technology, we have been able to comparatively assess smoke exposure conditions between two very different exposure platforms. For example, using the Borgwaldt RM20S exposure system we observed a deposition range between 25.75-0.22 μg/cm^2^ for smoke concentrations tested [22]. In contrast, we have presented data here characterising particulate deposition using the Vitrocell VC 10 and associated QCM module; in this study we saw a deposition range over the same approximate exposure time of 8.65-0.72 μg/cm^2^ (at the recommended 5 ml/min/well vacuum). By comparing QCM deposited mass values between different whole smoke exposure systems we can observe and understand the overlap in dose delivered. The resulting data allows a direct comparison between two very different exposure systems, defined by different smoke delivery mechanisms. The Borgwaldt RM20S delivers smoke in a ratio of air (smoke:air, v:v) whereas the VC 10 delivers smoke in a constant diluting airstream (L/min) with an applied vacuum through the module (ml/min/well). This means that for the first time we can directly compare exposure characteristics between machines: as an example, under our exposure conditions we have highlighted that a 1:25 smoke dilution on the RM20S equates to approximately a 2.0 L/min dilution on the VC 10 (3.28 and 3.41 μg/cm^2^ respectively). This comparison clearly shows how the QCM dose tool will enable future comparisons between different whole smoke exposure systems and facilitate the interpretation of biological responses from different platforms with more ease. This cross-comparison aids in the characterisation and confidence we have in the QCM dose tool itself. Furthermore, this approach could be applied to almost any whole smoke based exposure system or other aerosol generator (where the aerosol is consistent with tobacco smoke: spherical liquid droplet particles) which will only strengthen biological data in the future. This study also provided initial system characterisation of the VC 10, a technology where published information on its effectiveness as a whole smoke exposure system is only starting to appear [12,15].

## Materials and methods

### VC 10 set-up and smoking

In this study, dose was quantified from smoke generated by a Vitrocell VC 10 Smoking Robot. The VC 10 comprises of a rotary smoking carousel enclosed in a plastic ventilation hood. Mainstream smoke is drawn via the piston which sits under the carousel and delivers an aliquot of smoke to the dilution system. The dilution system is comprised of individual dilution bars where diluting air is added above and below the mainstream smoke. In this way, discontinuous smoke is added to continuous airflow, and diluted smoke travels the length of the dilution bar due to this continuous flow. The Vitrocell exposure modules dock under the dilution bar and smoke is drawn through the module onto the QCM (or cell cultures) via a vacuum; smoke not sampled through the module exhausts from the dilution bar (Figure 1).

The module lid has specially designed (trumpet/funnel) inlets for optimal aerosol distribution and particle deposition. All four smoke inlets dock to the dilution bar of the VC 10 during exposure. Integrated with the lid is the aerosol outlet which is attached to a vacuum pump via vacuum valves. The rate of the vacuum can be adjusted to change the amount of diluted smoke which is sampled from the dilution bar into the module. A QCM unit can easily be installed into the well where a cell culture insert would be exposed, simply by removing the island in the well and screwing it into the plug in the base; microbalance units can be installed into one, any or all of the positions 1–4 of the module. For a mammalian cell culture exposure at the ALI, the medium is supplied individually for each of the 4 well compartments and does not transit between them. Fresh medium exchange can be performed on a continuous basis per well compartment using a precision medium pump. If required, constant temperature of the unit is assured by a regulated flow of temperature controlled water through the module lid and base (Figure 2B).

In this study, the Vitrocell VC 10 Smoking Robot (serial number VC 10/141209) (Vitrocell® Systems, Waldkirch, Germany) (Figure 1) generated and diluted whole smoke from 9.4 mg 3R4F Kentucky reference cigarettes (University of Kentucky, Kentucky, USA). For all experiments, the VC 10 smoked for 24 minute duration at ISO 3308:2000 smoking regime (35 ml puff over 2 seconds, once a minute, to a defined number of 8 puffs from 3 cigarettes).

Cigarettes were smoked in *single mode*: only one cigarette was loaded into the rotary carousel and smoked at a time, thus the three cigarettes were smoked one after the other (this is in contrast to VC 10 *serial mode* smoking where up to 10 cigarettes can be smoked consecutively on the carousel). Once the 100 ml volume piston of the VC 10 had taken a puff from the lit cigarette (Figure 1, position 2) it delivered the 35 ml aliquot to the dilution system of the VC 10 over 8 seconds (without any resident hold time in the piston). After transit from the piston the whole smoke was diluted with 2 perpendicular jets of continuous purified laboratory air (Figure 1, position 3). Within the dilution system (Figure 1, position 4) the smoke jet through which the mainstream smoke was delivered was 2.0 mm ø, and the diluting air jets were 1.0 mm ø. Smoke doses were obtained by adjusting the diluting airflow delivered to the dilution system. Airflows selected for testing were 0.5, 1.0, 2.0 and 4.0 L/min and were set and maintained during the duration of the experiment by mass flow controllers (Analyt-MTC GmbH, Mülheim, Germany).

### QCM module

The 6/4 CF Stainless exposure module (Vitrocell® Systems, Waldkirch, Germany) had four identical QCM units installed into each separate well (Figure 2) and was docked under the VC 10 dilution system (Figure 1, position 5). Smoke was sampled from this stream of diluted smoke at various vacuum rates through the module. Vacuum rates selected for testing were 5, 10, 50 and 100 ml/min/well and were set and maintained during the duration of the experiment by vacuum valve blocks (Vitrocell Systems, Waldkirch, Germany) having been confirmed by mass flow meters (Analyt-MTC GmbH, Mülheim, Germany). Vacuum was generated by a Labport pump (KNF Neuberger Inc., Freiburg, Germany) which was attached to the 4 vacuum valve blocks, which in turn were attached to the 4 independent ports of the module lid (Figure 2B, position 2). To restrict particulate matter/tar being drawn via the vacuum into the vacuum valve blocks and pump, microfiber filters (Hirschmann Laborgeräte GmbH & Co. KG, Eberstadt, Germany) were installed into each of the 4 vacuum lines after the module (Figure 1, position 6).

The QCM units [36] and associated software (Vitrocell Systems, Waldkirch, Germany) utilised for quantification of smoke particle deposition *in vitro* have been previously published [22]. All QCMs read at a resolution of 10 nanograms/cm^2^/second, and mass readings were taken every 2 seconds during exposure and reported as mass per unit area. QCMs were allowed to stabilise (zero point stability) and plateau before and after exposure respectively. After the third (and final) cigarette was removed and extinguished, the four QCMs were left to record real-time data until all smoke particles in the module had fully deposited and mass values were observed to plateau, usually taking an additional 60–120 seconds, due to the application of a vacuum flow applied through the module.

### Statistics and graphics

All experiments were repeated 3 times; however deposition quantification experiments conducted at all airflows at 5 ml/min/well vacuum were replicated 5 times (Figure 3). All charts (Figures 3 &4) were produced using Minitab® version 16.1.0. Means of deposited mass ± standard deviation (Table 1) were calculated from the raw data in Microsoft Excel® 2010. Statistically significant differences in deposition across/between module positions (Table 2) were determined by one-way analysis of variance (ANOVA) with Tukey method and a confidence level of 95.0 in Minitab® version 16.1.0.

## Conclusions

In summary, we have characterised smoke particulate deposition within our VC 10 exposure system under varying vacuum flow rates and airflows following a 24 minute exposure to cigarette smoke (Table 1). This information will become invaluable in conceiving and designing future exposure scenarios. In addition the QCM tool has the added benefit of quantifying particle deposition in real-time. The QCM can be operated easily by a single user thus there is little requirement for technical support, consumables or analytical resource. Finally, we have demonstrated that the QCM can be used primarily for dose range finding investigations. However, we have also used the tool significantly for quality control (QC) of machines and set-up, for troubleshooting and also as an investigative tool for various improvements to the system or for variables (such as vacuum rate) which can be altered.

## Abbreviations

ALI: Air-liquid interface; ANOVA: Analysis of variance; ISO: International Organisation for Standardisation; QC: Quality control; QCM: Quartz crystal microbalance; SD: Standard deviation; VC 10: Vitrocell VC 10 Smoking Robot

## Competing interest

All authors are employees of British American Tobacco.

## Authors’ contributions

JA and DT conceived and designed the study. JA performed the experimental work, conducted the data analysis and statistics, and drafted the manuscript. DT reviewed the data collection and data analysis. DT, AD, DD and CM provided scientific support and reviewed the manuscript. All authors read and approved the final paper.
